# Novel Therapeutic Opportunities for Neurodegenerative Diseases with Mesenchymal Stem Cells: The Focus on Modulating the Blood-Brain Barrier

**DOI:** 10.3390/ijms241814117

**Published:** 2023-09-14

**Authors:** Pablo Vargas-Rodríguez, Alejandro Cuenca-Martagón, Julia Castillo-González, Ignacio Serrano-Martínez, Raúl M. Luque, Mario Delgado, Elena González-Rey

**Affiliations:** 1 Institute of Parasitology and Biomedicine Lopez-Neyra (IPBLN), CSIC, PT Salud, 18016 Granada, Spain; pablo.vargas@ipb.csic.es (P.V.-R.); juliacastillo@ipb.csic.es (J.C.-G.); ignacioserrano23@ipb.csic.es (I.S.-M.); mdelgado@ipb.csic.es (M.D.); 2 Maimonides Biomedical Research Institute of Cordoba (IMIBIC), 14004 Cordoba, Spain; alejandro.cuenca@imibic.org (A.C.-M.); bc2luhur@uco.es (R.M.L.); 3 Department of Cell Biology, Physiology, and Immunology, University of Cordoba, 14004 Cordoba, Spain; 4 Reina Sofia University Hospital (HURS), 14004 Cordoba, Spain; 5 CIBER Physiopathology of Obesity and Nutrition (CIBERobn), 14004 Cordoba, Spain

**Keywords:** blood-brain barrier, mesenchymal stem cell, neurodegenerative diseases, Alzheimer’s disease, neuroinflammation, Parkinson’s disease, multiple sclerosis, stroke

## Abstract

Neurodegenerative disorders encompass a broad spectrum of profoundly disabling situations that impact millions of individuals globally. While their underlying causes and pathophysiology display considerable diversity and remain incompletely understood, a mounting body of evidence indicates that the disruption of blood-brain barrier (BBB) permeability, resulting in brain damage and neuroinflammation, is a common feature among them. Consequently, targeting the BBB has emerged as an innovative therapeutic strategy for addressing neurological disorders. Within this review, we not only explore the neuroprotective, neurotrophic, and immunomodulatory benefits of mesenchymal stem cells (MSCs) in combating neurodegeneration but also delve into their recent role in modulating the BBB. We will investigate the cellular and molecular mechanisms through which MSC treatment impacts primary age-related neurological conditions like Alzheimer’s disease, Parkinson’s disease, and stroke, as well as immune-mediated diseases such as multiple sclerosis. Our focus will center on how MSCs participate in the modulation of cell transporters, matrix remodeling, stabilization of cell-junction components, and restoration of BBB network integrity in these pathological contexts.

## 1. Introduction

The evidence indicates that longevity is currently increasing worldwide, and the occurrence of cumulative disabilities is the price to pay for living longer [1]. It is widely recognized that aging is a natural, progressive, and inevitable process that occurs in all organisms, although the functional and morphological changes affecting the body tissues and organs during its progression are highly variable [2]. Specifically, aging-associated changes in the central nervous system (CNS) are of crucial relevance, and the prevention and treatment of neurodegenerative conditions represent one of the greatest challenges for modern societies. However, therapeutic options to treat CNS-related disorders are very limited, mainly due to the fine-tuned status of the brain, the complexity of neurological diseases, and the lack of knowledge of their etiology and pathophysiology.

An additional challenge in this scenario comes from the singular presence in the CNS of the blood-brain barrier (BBB), a complex, dynamic, and structured network of cells and proteins responsible for protecting the brain and regulating the transport of substances and cells from the peripheral circulation to the CNS. Because keeping the integrity of the BBB is critical for maintaining a constant environment in the CNS in healthy conditions, investigating how this structure changes during aging, and specifically in pathological conditions, has lately received special attention worldwide. Although some researchers have demonstrated minor and variable BBB leakage in healthy aging without immune infiltration and neuropathological signs [3], evidence indicates that BBB integrity is compromised in most neurological disorders, including those associated with aging [4,5,6,7,8,9,10,11]. Therefore, BBB disruption emerges as a key innovative element to design new therapeutic approaches to treat neurodegeneration during aging and neurological afflictions.

Recently, stem cells isolated from adult mesenchymal tissues (MSCs) have emerged as attractive candidates for the treatment of aging-associated neurological diseases [12,13]. While many studies have explored the involvement of the neuroprotective, neurotrophic, and immunomodulatory capacities of MSCs in their therapeutic actions in neuroinflammatory and neurodegenerative disorders, the potential additional role played by MSCs in improving the sealing and modulation of the BBB has scarcely been addressed. 

This review aims to examine the structure and functions of the BBB and how its impairment, along with changes in transporters, extracellular matrix, and cell-junctional components, influences the onset and progression of several neurodegenerative disorders. We will specifically focus on four neurodegenerative conditions, namely Alzheimer’s disease (AD), Parkinson´s disease (PD), multiple sclerosis (MS), and stroke. While AD, PD, and MS are chronic diseases with etiologies that remain mostly unknown, stroke is mainly recognized as an acute pathological state of the brain with well-described pathogenesis and etiology. Although these disorders exhibit different clinical and pathological signs, they all progress with neuroinflammation and neurodegeneration, are significantly associated with aging, and display profound alterations in cerebral vasculature and microvessel components. Importantly, they all respond positively to MSC-based therapies. Our review will also delve into the molecular mechanisms involved in the treatment of neurological pathologies with MSCs, with special attention to their ability to restore the integrity of the BBB. Finally, we will discuss how to further improve MSC therapies by specifically targeting the BBB. 

## 2. Structure and Role of the Blood-Brain Barrier 

**Figure 1:**
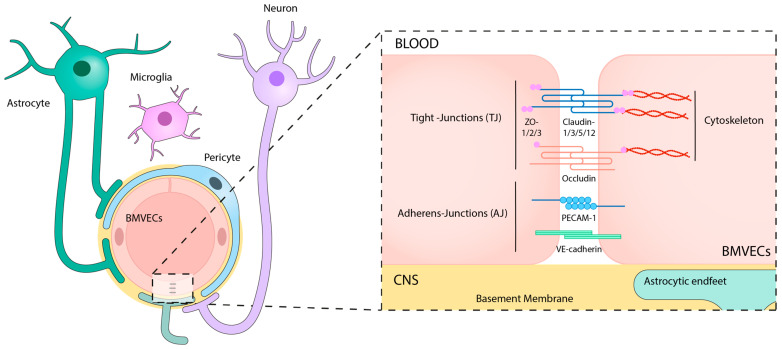
Schematic representation of the blood-brain barrier (BBB). It is located within the neurovascular unit (NVU) and constituted by endothelial cells interconnected by tight junctions (TJ) and adherens junctions (AJs), and neighboring cells, such as pericytes, astrocytes, neurons, and microglia. CNS: central nervous system; BMVECs: brain microvascular endothelial cells; ZO: zonula occludens; PECAM: Platelet endothelial cell adhesion molecule; VE-cadherin: vascular endothelial cadherin.

The BBB is a complex and dynamic microvascular structure composed of several types of cells, which maintains the homeostasis of the CNS by regulating the supply of molecules and filtering potentially harmful compounds from the bloodstream to brain tissues and back [14]. Its main scaffold is structured into a neurovascular unit (NVU) that is shaped by a tight layer of brain microvascular endothelial cells (BMVECs) surrounded by astrocyte end-feet and pericytes, all of them embedded in an extracellular matrix network and the basement membrane [15] (Figure 1).

The BMVECs are highly specialized endothelial cells that show unique structural and biological properties compared to peripheral endothelial cells [16], including low pinocytic activity, absence of fenestration, low levels of leukocyte adhesion molecules, high expression of intercellular junctions, mainly tight junctions (TJs) and adherens junctions (AJs) [17,18], and increased number of mitochondria to supply the energy that is required for the active transendothelial transport of molecules [19]. Additionally, BMVECs regulate BBB permeability by mainly controlling the intercellular and intracellular transport of cells and molecules via cellular junctions and specific membrane carriers, channels, and transporters [20]. Moreover, these cells present a very remarkable apicobasal polarity, based on a differential membrane composition (lipids, glycoproteins, receptors, and transporters) between the luminal and the abluminal sides [21]. For instance, enzymes like γ-glutamyl-transpeptidase [22] or alkaline phosphatases [23] are located in the luminal face of the endothelium, while Na^+^-K^+^ ATPase [24] and the Na^+^-dependent neutral amino acid transporter [25] are present at the basal membrane. 

BMVECs are wrapped by pericytes which contribute to the regulation of endothelial cell proliferation, survival, migration, differentiation, vascular branching, and blood flow control [26]. Smooth muscle cells are also found around large vessels (arteries, arterioles, venules, and veins), providing strength and elasticity, and playing a significant role in basal tone maintenance, blood pressure, and blood flow distribution [27,28]. Pericytes are localized along capillaries and embedded within the BM. They regulate blood flow, modulate immune and phagocytic responses after brain injury, and promote angiogenesis in the adult CNS [29,30].

Astrocytes are the major glial cell that enfolds the endothelium of the BBB [31]. They connect to BMVECs through their end-feet, contributing to BBB consistency and determining its properties [30]. Astrocytic end-feet contain a set of proteins that interact with the vascular tube, such as dystroglycan-dystrophin complex or aquaporin 4 (AQP-4), among others. The latter is critical for regulating water homeostasis in the CNS, while the dystroglycan-dystrophin complex links the astrocytic skeleton to the BM [32]. Since astrocytes serve as the cellular linkage between the neuronal circuitry and the vascular system in the CNS, they release signals that regulate the blood flow in response to neuronal activity. For instance, they control the contraction and dilation of SMCs and pericytes [33]. Additionally, they contribute to the formation of endothelial cell TJs through vascular endothelial growth factor (VEGF)-mediated signals [34] and regulate tissue inhibitor metalloproteinases (TIMPs), which maintain the balance between deposition and degradation of the extracellular matrix components [35].

The basement membrane (BM) is a highly organized sheet composed prominently of extracellular matrix proteins (collagen IV, laminins, nidogen, and perlecan). It plays an important role in providing structural support, cell anchoring, and signaling transduction [36]. Two types of BM of the BBB have been characterized: the inner vascular BM, secreted by BMVECs and pericytes, which contains laminins α1 and α2; and the outer parenchymal BM, secreted by astrocytes, which contains laminins α4 and α5 [37,38]. Nevertheless, the BM is largely understudied, in comparison to the cellular components of the BBB, probably due to its intrinsic complexity and the lack of research tools [39].

The exchange of molecules and cells across the BBB requires specific transporters, channels, and receptors. Two major mechanisms, named paracellular and transcellular transport, have been identified. Table 1 describes some of the main transporters. Paracellular transport is the main pathway used for the exchange of small hydrophilic substances. On the other hand, the transcellular transport involves the movement of molecules through the cell membrane of BMVECs. It occurs via several mechanisms depending on the nature of the molecule, such as passive diffusion, facilitated diffusion, active transport, and receptor-mediated transport. Importantly, gases and small lipophilic molecules do not require transporters to cross the BBB and freely diffuse across the endothelium [40]. 

Both paracellular and transcellular processes are precisely regulated by junctional structures, mainly TJs, with some contribution from gap junctions and AJs [41]. TJs are intricate structures located along the membranes of adjacent BMVECs, intermingled with AJs, providing stability and consistency to the BBB [42,43] (Figure 1). Among TJ proteins, claudin-1, -3, -5, -12, and occludin control the transportation of solutes and ions [44]. These proteins are associated with the cytoskeleton, primarily based on actin and vinculin, through scaffolding proteins, such as ZO-1, -2, and -3. Moreover, dystrophin acts as a scaffold protein that mobilizes actin and vinculin proteins [45]. AJs create inter-endothelial contact connections, maintained by proteins such as VE-cadherin and platelet endothelial cell adhesion molecule-1 [46,47] (Figure 1), that contribute to the continuous crosstalk with TJs for paracellular transportation. Similarly to TJs, AJs are attached to the cytoskeleton, contributing to the regulation of cellular transportation of lymphocytes, monocytes, or neutrophils [48,49,50]. Pericyte-endothelial junctions also contain cadherins, with N-cadherins forming homophilic interactions between pericytes and BMVECs, thereby maintaining vascular integrity [51]. Furthermore, BMVECs interact with the BM, establishing AJs via α- and β-integrin receptors, which are transmembrane glycoproteins involved in the extracellular matrix connection to the endothelial cytoskeleton [44]. Conversely, gap junctions serve as intercellular channels facilitating cytoplasmic connections between neighboring cells, enabling selective communication of molecules primarily dependent on molecular size, driven by passive diffusion [52]. In the brain, endothelial cells express the gap junctions connexin 37 (Cx37), Cx40, and Cx43 [53], while astrocytes express Cx30 and Cx43 [54]. In addition to their channel function, certain connexins also play a regulatory role in the expression of other junctional molecules, such as Cx43 interacting with N-cadherin [55]. 

**Table 1:** Proteins involved in transcellular transportation in BMVECs of the BBB.

Transporter	Cargo	Location	Description	Source
Glucose Transporter 1 (GLUT-1)	Glucose	Abluminal and luminal side	Main glucose transporter of BMVECs. Also expressed in astrocytes but not in neurons. Na^+^ dependent transporters	[40,56]
Large neutral amino acid transporter 1 (LAT1)	Large neutral amino acids	Abluminal and luminal side	Abluminal side LAT1 transport is dependent of Na^+^ concentration. Bidirectional transport	[40,57]
Cationic amino acid transporter 1 and 3 (CAT1/3)	Cationic amino acids	Abluminal and luminal side	CAT-1 is pH and Na^+^ independent but sensitive to changes in membrane potential	[58]
Na^+^-dependent transporters for glutamate exist on astrocytes 1 and 2 (EAAT1/2)	Glutamate	Abluminal side	Expressed in astrocytes. Possible protective mechanism against glutamate neurotoxicity	[59]
Monocarboxylate transporters (MCT1)	Monocarboxylic acids (lactate, pyruvate, and acetoacetate and β-hydroxybutyrate)	Abluminal and luminal side	Intracerebral transport. Located in BMVECs and astrocytes. The transport mechanism is a H^+^ cotransporter or a monocarboxylate exchanger	[60,61]
Insulin receptor (IR)	Insulin	Abluminal and luminal	Located in BMVECs. Insulin binding activates IR by phosphorylation of beta-chain region. Impaired phosphorylation response in AD	[62]
Low-density lipoprotein receptor–related protein 1 (LRP1)	APO2 and APO3	Mainly in the abluminal side	Located in BMVECs. LRP1 binds to Aβ aggregates and mediates their clearance from brain to blood. LRP1 level diminished in AD patients leads to aggregates accumulation	[26,63]
Receptors for advanced glycation end-products (RAGE)	Advanced glycation end products (AGE), high mobility group box-1 (HMGB-1) protein	Mainly at the luminal side	Located in BMVECs, microglia, and astrocytes. Upregulated in AD. It mediates the influx of Aβ into the brain	[64,65]
P-glycoprotein, ATP-binding cassette 1 (P-gp, ABCB1)	Xenobiotics and drugs	Expressed in the luminal side	P-gp is a unilateral efflux pump from blood to brain. It uses ATP in the active transport of substances. It is crucial in the ADMET properties of pharmaceutical drugs. In AD, P-gp is involved in accumulation of Aβ peptides in the CNS	[66,67]
Transferrin receptor protein (Tfr)	Transferrin (apo- and holo-transferrin)	Abluminal and luminal side	Primary iron transporting system. Highly enriched in BMVECs. Studied as a targeted transporter of therapeutics to the brain	[68]

## 3. MSCs as a Therapeutic Option in CNS Disorders

Despite notable advancements in the management of symptoms accompanying the neurodegeneration/neuroinflammation in AD, PD, MS, and in the development and progression of acute stroke, with treatments that enhance quality of life and increase lifespan, the available drugs only slow the progression of neuronal death. Given the multifactorial and complex nature of these conditions, the primary causal agent remains unclear, and it is imperative to develop multi-target therapies that address the different causes/consequences of these disorders, such as neuroinflammation, neuronal cell death and dysfunction, and BBB disruption.

MSCs are emerging as one of the most promising cell therapies against different immune-mediated diseases due to their unique properties. MSCs are multipotent cells able to differentiate into mesodermal lineages (fibroblast, osteocyte, adipocyte, and chondrocyte) and, in some cases, into endodermal or ectodermal (neuronal) fates [69]. The scarce expression of the major histocompatibility complex and other co-stimulatory molecules makes MSCs immune-privileged cells. This immune status allows MSCs to be used in an allogenic manner without requiring additional immunosuppression [70].

The International Society for Cellular Therapy has defined MSCs based on their expression of CD90, CD73, CD105, and CD44, while lacking the expression of CD45 and CD31 [71]. These markers help to distinguish MSCs from other cell types and are used to identify and isolate these cells for research and therapeutic purposes. 

In adults, several tissues act as MSC reservoirs [72]. The first type of MSC to be described were bone marrow-derived mesenchymal stem cells (BM-MSCs) [73], making BM the primary source for MSC isolation. However, the process of obtaining BM-MSCs involves a highly invasive and painful procedure that requires anesthesia, posing a risk of infection [74]. Alternatively, adipose tissue-derived MSCs (ASCs) can be isolated from biological material generated during liposuction or lipectomy after medical interventions. The natural abundance of MSCs in adipose tissue, which is approximately 500 times higher than in BM, accompanied by easier isolation, has led to an increased utilization of ASCs [75]. Additionally, a recent study has demonstrated that ASCs exhibit lower immunogenicity and transcriptomic heterogeneity compared to BM-MSCs [76]. Apart from adult tissues, MSCs can also be derived from birth-associated tissues, such as the umbilical cord Wharton’s jelly (WJ). WJ-MSCs have emerged as an ideal source of MSCs for therapy due to several advantages: they can be harvested painlessly in abundance without causing donor site morbidity, are easy to isolate and culture, possess a high proliferative rate, and retain their stemness properties in vitro [77]. 

In the context of neurodegeneration, there is growing interest and promise in therapies based on MSCs. As of July 2023, 249 clinical studies were found on www.clinicalstrials.gov [78] with the terms “Nervous System Diseases” and “Mesenchymal Stem Cell”, reflecting the potential of these cells in addressing neurodegenerative/neuroinflammatory disorders. A summary of studies focused on AD, PD, MS, and stroke (a total of 96 from the 249 trials) identifying the tissue for MSC isolation, donor, route of administration, and pathological target is shown in Table 2.

The main mechanisms exerted by MSCs that contribute to their potential efficacy include:

- Neuroprotective effect: MSCs have demonstrated to have an important neuroprotective effect, as they secrete neurotrophic growth factors such as glial cell-derived neurotrophic factor, VEGF, brain-derived neurotrophic factor, and nerve growth factor (NGF) [79], as well as anti-apoptotic factors like Bcl-2 [80]. These factors enable MSCs to promote nervous regeneration, inhibit neuronal apoptosis, and induce endogenous neurogenesis. For example, Oh et al. [81] demonstrated that intravenous injection of MSCs increased hippocampal neurogenesis and differentiation of neural progenitor cells into mature neurons in Aβ-treated mice (AD model) by augmenting the Wnt signaling pathway. Additionally, MSCs may inhibit stroke-associated apoptosis through the Bcl-2 pathway in neurons and astrocytes from rats [82]. Furthermore, MSCs can transfer healthy mitochondria to damaged cells, protecting neural stem cells from neurotoxic agents. MSCs may transfer this organelle in various ways, including gap junctions, cell fusion, microvesicles, and through tunnelling nanotube formation [83]. Mitochondria play a crucial role in maintaining metabolic homeostasis, and defects such as membrane leakage, electrolyte imbalances, activation of pro-apoptotic pathways, and mitophagy have been implicated in the pathogenesis of various CNS disorders [84]. It has been demonstrated that the ability of MSCs to transfer healthy mitochondria to damaged cells protects neural stem cells from neurotoxic agents [85], and has garnered significant attention in the field of cellular therapy for CNS disorders;
- Immunomodulatory role: MSCs can interact with the immune system and participate in both innate and adaptive immunity due to their significant immunoregulatory functions. This indicates that, depending on the environment in which MSCs are introduced, they can modulate the response. Thus, in an inflammatory environment, MSCs exhibit anti-inflammatory behavior. By expressing different molecules such as transforming growth factor β, indoleamine 2,3-dioxygenase, prostaglandin E2, nitric oxide, and interleukin-10 (IL-10), they can interact with immune cells either through direct cell-to-cell contact or via paracrine activity [86,87,88,89,90]. MSCs can also modulate the macrophage/microglia polarization, upregulating the ratio of anti- versus pro-inflammatory responses [91], suppress Th1 and Th17 responses, enhance the maturation of DCs from monocytes, and enhance the Th2 response and the generation of Forkhead Box P3 positive Treg population. Moreover, some studies reported that the secretion of IL-6 by MSCs can inhibit astrocyte apoptosis, increase the neuroprotective population of astrocytes, and reduce neuron damage post-injury [92];
- Regulation of protein clearance: treatment with MSCs has been shown to induce the secretion of neprilysin in vitro and in vivo, improving the endogenous machinery for the degradation of Aβ-plaques and enhancing the clearance of these aggregates [93]. This is particularly relevant as abnormal protein aggregation is one of the major hallmarks of neurodegenerative diseases like PD and AD [94].

## 4. MSCs as Promising Modulators of the BBB in Neurodegenerative Disorders

As described before, while it remains unclear whether BBB disruption is a cause or a consequence of neuroinflammation, it is undoubtedly a crucial component of CNS pathologies. Unfortunately, the BBB is often viewed as a challenge that hinders the delivery of drugs to the CNS and reduces the efficacy of conventional treatment approaches for neurodegeneration. Therefore, the potential of pharmacological interventions targeting the BBB could represent a promising therapeutic strategy for the neuroinflammatory-mediated neurodegenerative diseases [12,13]. In addition to the neuroprotective and immunomodulatory roles of MSCs in neurodegeneration, recent reports have pointed out a beneficial effect of MSCs on modulating the disrupted BBB.

In general, the delivery of MSCs to the CNS is highly diverse, although systemic administration, particularly intravenous infusion, is the preferred method (Table 2). When MSCs are infused intravenously, they transiently accumulate in the lungs for 1–3 h, followed by a gradual movement to other tissues such as the liver, spleen, kidney, and bone marrow [95]. Interestingly, MSCs have shown the ability to reach brain vessels and adhere to them 6 h post-injection, according to Rüster et al. [96], thanks to specific interactions with endothelial cells through adhesion molecules such as P-selectin and VCAM-1/VLA-4. While some reports indicate that, after a middle cerebral artery occlusion (MCAO) model of stroke, injected MSCs accumulate in the vessels of the infarcted region [97,98], other studies describe no MSCs being detected in the cerebral parenchyma after an intra-arterial injection in an Alzheimer’s disease mouse model [99]. Therefore, although MSCs are capable of rolling and executing a coordinated extravasation through activated endothelia in other tissues, allowing them to access sites of damage [96], it remains unclear whether this cellular therapy can cross the BBB and exert its function within the brain tissue.

Nevertheless, despite their uncertain ability to cross the BBB and penetrate to the brain, MSCs can directly make contact with endothelial cells in the damaged area. This interaction likely enables MSCs to exert their paracrine functions to other cells of the NVU and the BBB from this location (Figure 1). In fact, the scientific community is currently exploring two interesting derivatives of MSCs: genetically modified MSCs and the use of MSC-derived extracellular vesicles (MSC-EVs). Genetically modified MSCs provide the opportunity to enhance the therapeutic effect of MSCs by improving their inherent functions or enabling them to synthesize drugs or active compounds. On the other hand, direct use of the secretome in the form of MSC-EVs improves penetration through the BBB. In the following sections, we will provide examples of these strategies and the cellular and molecular beneficial effects exerted by MSCs in preclinical models of neurodegenerative conditions characterized by a severe disruption of the BBB.

### 4.1. Alzheimer’s Disease 

Alzheimer´s disease (AD) is a progressive neurodegenerative disease characterized by a cerebrovascular and neuronal dysfunction, resulting in a gradual decrease in cognitive functions [100]. In 2019, it was estimated that 50 million people suffered from AD [101]. The principal pathological hallmarks are extracellular amyloid-β (Aβ) deposition and neuronal accretion of phosphorylated tau-forming neurofibrillary tangles [102]. Aβ is a proteolytic by-product derived from the amyloid precursor protein, produced by several cleavages via β- and γ-secretases. There are two main types of AD: early onset AD, which is a rare form affecting <1% of AD cases in subjects <65 years old and is caused by genetic mutations, and late onset AD, which is the most frequent form, and primarily affects to patients >65 years old. While genetic factors may potentially contribute to its development, specific mutations that directly cause an increase in proteolytic cleavage in patients have not been observed [103].

#### 4.1.1. Dysfunctional BBB in AD

**Figure 2:**
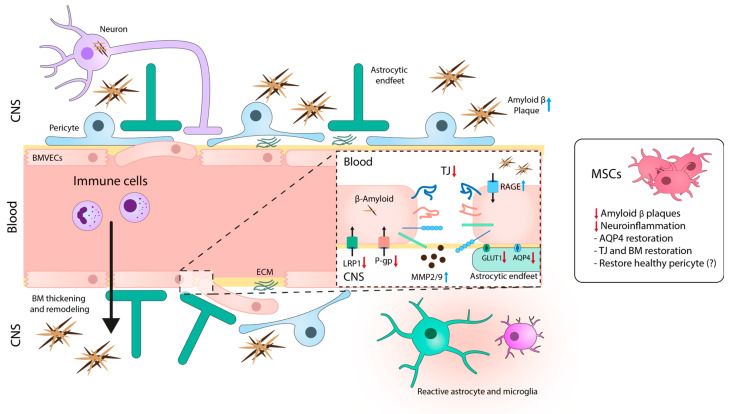
Blood-brain barrier (BBB) disruption in Alzheimer’s disease (AD) pathogenesis. The breakdown of the BBB in AD is characterized by a cascade of events, including loss of TJ integrity, disorganization of the basement membrane (BM) and extracellular matrix, pericyte degeneration and detachment, activation of glial cells, astrocyte depolarization, and alteration of BBB transporter expression (LRP1, P-gp, and GLUT1 are reduced, whereas RAGE expression is increased). These modifications may lead directly or indirectly to disturbed Amyloid β (Aβ) clearance in the neurovascular unit (NVU), contributing to further neuronal toxicity and AD pathogenesis. Treatment with MSCs restore BBB integrity by stabilizing TJ, BM, and extracellular matrix (ECM) remodeling, and reduces the neuroinflammation and Aβ accumulation. CNS: central nervous system; BMVECs: brain microvascular endothelial cells; BM: basement membrane; RAGE: receptor for advanced glycation end products; LRP1: Low density lipoprotein receptor-related protein 1; P-gp: permeability glycoprotein; GLUT1: glucose transporter 1; MMP: metalloproteinases; TJ: tight junctions; AQP: aquaporin. Blue and red arrows mean increase and reduction, respectively.

BBB dysregulation plays a significant role in the pathogenesis of AD, affecting various components of the NVU (Figure 2). 

For instance, the presence of Aβ disrupts the organization of TJs and AJs (i.e., occludin, claudin-5, and ZO-1) in BMVECs, leading to compromised barrier activity [104,105]. Additionally, the reduction in GLUT-1 in cerebral microvessels of AD patients contributes to vessel degeneration and further exacerbates the disease [106,107]. Conversely, patients with mild cognitive impairment, which is the precursor of AD, display increased BBB permeability that correlates with high levels of soluble platelet-derived growth factor receptor β in the cerebrospinal fluid, which is indicative of pericyte damage [108]. In fact, the decreased number of pericytes in AD patients may worsen the accumulation of Aβ both in brain parenchyma and blood vessels. 

On the other hand, astrocytes in AD patients showed reduced expression of AQP4 in perivascular end-feet and increased levels of astrocytic activation markers [109]. In fact, the accumulation of Aβ in the brain leads to pericyte degeneration and loss, a dysregulated BM, and astrocytic end-feet depolarization with loss of AQP4, which will decrease the Aβ clearance, fueling a pathogenic feedback loop. Thickening of the BM [110] and increased collagen levels in these structures [111,112] are also common features in AD patients. MMP2 and MMP9 are significantly activated in the NVU, contributing to BM remodeling in AD [113,114]. 

Furthermore, transporters of the BBB, such as RAGE, LRP1, or P-gp, are key elements in the regulation of Aβ clearance. In fact, analysis of microvessels and BMVECs in postmortem AD brains showed high expression of RAGE, which mediates Aβ entry into the brain [115], and reduced expression of LRP1 and P-gp, involved in the clearance of cerebral Aβ [116,117,118]. The immune system is also compromised in AD, and cells like monocytes, lymphocytes, or neutrophils can cross the BBB in response to Aβ accumulation and the augmentation of vascular adhesion molecules, contributing to the pathogenesis of AD [119,120,121].

#### 4.1.2. Therapeutic Opportunities for MSCs Targeting the BBB in AD

Currently there are no effective treatments to cure or slow AD progression. However, emerging evidence suggest that MSC therapy could be a promising approach. In general, MSC transplantation has been found to decrease Aβ deposits and plaques, and tau-related cell death in vivo. The paracrine effects of MSCs stimulate neurogenesis, synaptogenesis, and neuronal differentiation, demonstrating neuroprotective functions. Moreover, their immunoregulatory properties, which modulate microglia/astrocytes’ activity state, can deactivate neuroinflammatory responses via several transcription factor signaling pathways [122]. 

The BBB represents a major challenge in treating AD, and different studies have focused on the action of MSCs on cerebral vasculature (Figure 2). For instance, Garcia et al. [123] demonstrated the ability of intracerebrally transplanted MSCs in a 2xTg-AD mouse model to promote neovascularization in the hippocampus. Specifically, they found that MSCs genetically modified to express VEGF enhanced their therapeutic efficacy in promoting neovascularization. Focusing on transporters involved in AD, Son et al. [124] modified MSCs to express the secreted isoform of RAGE (sRAGE), which inhibits the interaction between RAGE and its ligands, thus preventing the adverse effects of this signaling pathway. When activated by Aβ oligomers, RAGE can lead to cell stress, generation of ROS, and RAGE-mediated inflammation and neurodegeneration. Transplantation of sRAGE-MSCs into 5xFAD transgenic mice reduced the deposition of Aβ, cell death, and inflammation. 

In a rat model of cerebral small vessel disease, a pathology characterized by Aβ deposition equivalent to AD, the intravenous infusion of MSCs restored the polarity/distribution of AQP4 to the end-feet of astrocytes, relieving cerebral edema and promoting the clearance of Aβ [125]. In another study, Tachibana et al. [126] implanted mouse MSC-derived pericytes into the brains of APP/PS1 mice and observed a reduction in Aβ levels in the hippocampus, an effect that was mediated by LRP1. Interestingly, a recent study has shown that, in a model of microfluidic BBB-like microvasculature, BM-MSCs emulate more efficiently the function of perivascular pericytes than induced pluripotent stem cell-derived pericytes, leading to greater restoration of TJs and the abluminal BMs [127]. In fact, there are several similarities between MSCs and pericytes. Pericytes express a similar pattern of immunological markers (CD44, CD90, CD73, CD105, and CD45), are self-renewable, and have the capacity to differentiate into nervous cells, mainly glial cells, in vivo [128]. Therefore, MSCs could potentially supply the loss of pericytes in AD. In summary, these results highlight the need for further research in this field, as understanding the role of MSCs in modulating the BBB in the context of AD is essential to develop effective therapies.

### 4.2. Parkinson’s Disease 

Parkinson´s disease (PD) is the second most common neurodegenerative disease after AD. This progressive disorder is characterized by the loss of dopamine neurons in the substantia nigra pars compacta (SNpc) and the accumulation of filamentous and oligomeric inclusion bodies (Lewy bodies) composed of misfolded α-synuclein proteins. These structures disrupt cellular processes and lead to neuronal degeneration, mainly leading to motor dysfunction. Its etiology is still unknown and current treatments are mainly focused on symptoms [129]. 

#### 4.2.1. Dysfunctional BBB in PD

**Figure 3:**
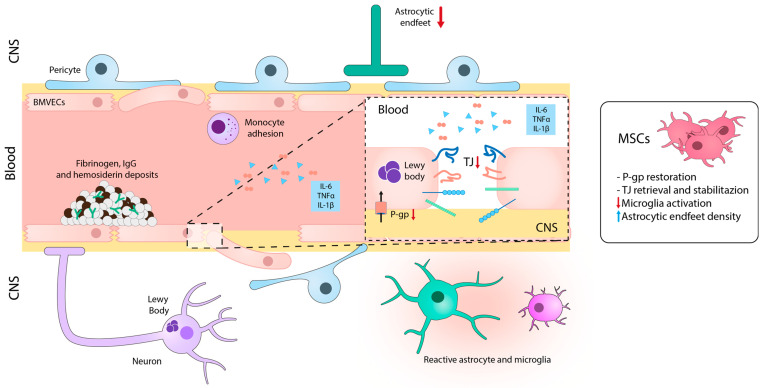
Schematic image illustrating blood-brain barrier dysfunction in PD. Alterations in BMVECs are characterized by decreased TJ and AJ proteins and disorganization of the basement membrane. In addition to the reduction in astrocytic end-feet and pericyte loss, these changes can lead to BBB breakdown and subsequent accumulation of fibrinogen, thrombin, and hemosiderin, which together with α-synuclein can activate glial cells and injure dopaminergic neurons. Treatment with MSCs recovers BBB integrity by stabilizing the TJ structure and decreases the production and accumulation of neuroinflammatory and neurotoxic mediators. CNS: central nervous system; BMVECs: brain microvascular endothelial cells; TJ: tight junctions; P-gp: permeability glycoprotein; Ig: immunoglobulin. Blue and red arrows mean increase and reduction, respectively.

Although decades ago, NVU was not recognized as an important element of the pathogenesis of PD, it is now well-established that BBB disruption is associated with PD (Figure 3). 

Nevertheless, further research is needed to completely understand the molecular mechanism of BBB disruption in this context [130]. Brain tissue from PD patients showed perivascular deposits of fibrinogen or fibrin, IgG, and hemosiderin in specific regions, indicating BBB disruption [131,132,133]. In fact, degeneration of BMVECs and TJs, as well as disorganization of the components of the BM, have been reported in PD brain tissues [132]. In addition, patients with idiopathic PD show genetic mutations that affect NVU components. For example, mutations in leucine-rich repeat kinase 2 in BMVECs lead to increased monocyte attachment in PD patients [134]. Moreover, several PD patients have mutations in the *MDR1* gene, which encodes P-gp in BMVECs, resulting in reduced pump function [135]. 

Angiogenesis is also affected in PD. Although PD patients show augmented vascular density in the SNpc, in the proximity of neuronal damage, these new microvessels display impaired maturation processes and altered diameters [136]. While new microvascular architecture may allow the supply of nutrients and cellular debris, it also raises the risk of leakage and infiltration of toxins, drugs, and immune cells, potentially exacerbating the pathology [137,138]. Moreover, proinflammatory cytokines secreted by activated immune and glial cells (such as TNF-α, IL-1β, and IL-6) [139] can decrease the expression of ZO-1 and occludin, leading to a disruptive state of TJs and subsequently contributing to BBB breakdown [140].

#### 4.2.2. Therapeutic Opportunities for MSCs Targeting the BBB in PD

Over the last decade, preclinical studies investigating the potential of MSCs in the context of PD have been performed (Figure 3). For instance, Chao et al. conducted an extensive study on the effect of MSCs using the preclinical model of PD induced by 1-methyl-4-phenyl-1,2,3,6-tetrahydropyridine (MPTP) [141]. They found that MSCs, intraperitoneally infused 24 h after the last MPTP injection, migrated to the SNpc and efficiently rescued dopaminergic TH+ neurons. Moreover, the treatment with MSCs restored BBB integrity, as indicated by the retrieval of TJ-protein expression (claudin-1, claudin-5, and occludin). 

Other studies have recently demonstrated that treatment with MSC-derived exosomes regulates genes associated with the angiogenesis of human BMVECs in vitro, resulting in increased expression of *Angpt1* and *Flk1*, as well as the secretion of the intercellular adhesion molecule 1 (ICAM1) protein. Injecting these exosomes into an MPTP-induced PD model resulted in their homing to the injured brain and a significant recovery from the disease. Additionally, there was an increase in the expression of ICAM1 and CD31 markers in the striatum and SNpc [142]. Conversely, in an LPS injection model into the SNpc, treatment with MSCs increased the expression of P-gp in endothelial cells and restored BBB integrity [143]. The study suggested that MSCs decreased the proinflammatory activation of microglia and modulated the VEGF-A signaling through astrocytes, leading to an increase in the astrocytic end-feet density. This process stabilized the expression of TJ proteins. These results suggest the relevance of modulating the BBB in PD for developing effective therapies against this debilitating disease.

### 4.3. Multiple Sclerosis

Multiple sclerosis (MS) is an inflammatory neurodegenerative autoimmune disease that affects the CNS. In MS, the immune system generates a complex response against myelin sheaths that wraps nerve axons, eventually leading to inflammation, demyelization, axonal degeneration, and ultimately neuronal loss [144]. Immune cells cross the damaged BBB and release proinflammatory cytokines, such as TNF-α, IFN-γ, or IL-17, which directly attack myelinating oligodendrocytes [145] or provoke a pro-inflammatory polarization of microglia and astrocytes that finally causes oligodendrocytes loss [146]. 

There are different types of MS depending on the evolution of the disease: relapsing-remitting MS (RRMS) and primary/secondary progressive MS (PPMS/SPMS). RRMS is characterized by alternating periods of symptom enhancement (relapses) and partial or complete recovery of neurologic function (remissions). Conversely, progressive MS (15% of MS patients) is marked by a gradual worsening of symptoms without periods of relapses or remissions. On the contrary, SPMS follows the initial relapsing-remitting course [144]. 

#### 4.3.1. Dysfunctional BBB in MS

**Figure 4:**
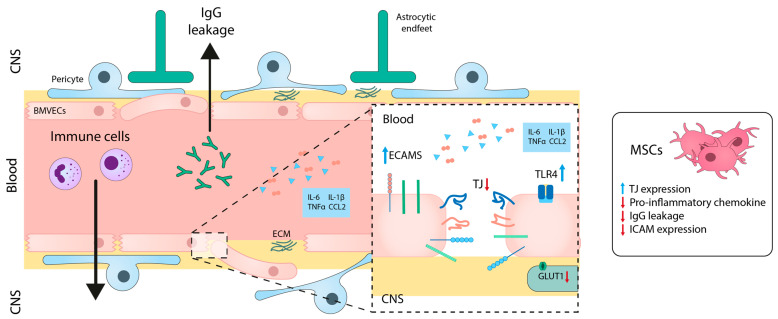
Blood-brain barrier alterations in multiple sclerosis. Hallmark features of MS development include an early BBB breakdown accompanied by reduced TJ expression, endothelial degeneration, leukocyte infiltration, and neuroimmune activation. Treatment with MSCs exerts immunomodulatory and neuroprotective roles in MS that in turn involve the stabilization of BBB integrity. CNS: central nervous system; ECM: extracellular matrix; BMVECs: brain microvascular endothelial cells; ECAMs: endothelial cell adhesion molecules; ICAM: intercellular adhesion molecules; TJ: tight junctions; TLR: Toll-like receptor; GLUT1: glucose transporter; Ig: immunoglobulin. Blue and red arrows mean increase and reduction, respectively.

Although the etiology of the disease is not completely understood, a vast body of evidence suggests the importance of BBB disruption in the pathology of MS (Figure 4). 

Inflammation affects several components of the NVU and hampers the physiologic function of numerous transport mechanisms. BBB permeability may be an important early step that is correlated to the initiation of a CNS-specific immune response [147]. Pro-inflammatory mediators, such as IL-1β [148], IL-6 [149], TNF-α [150], and chemokine (C-C motif) ligand 2 (CCL2) [151], reduce the expression of TJ and AJ proteins in different in vitro BBB models. Specifically, the expression of Toll-like receptors, which play a significant role in modulating MS, is significantly increased in BMVECs in response to ROS and TNF-α [152,153]. The expression of other transporters, like GLUT-1 [154], LAT1 [155], or P-gp [156], is also affected by the exposure of BMVECs to inflammatory mediators. Moreover, pro-inflammatory molecules can cause pericyte detachment from BMVECs and undergo transformation into phagocytic or fibroblastic-like cells [147]. Regarding the autoimmune component of MS, activation of BMVECs with Th1 cytokines (IL-2, TNF-α, IFN-γ) modulates the BBB phenotype and stimulates the expression of endothelial cell adhesion molecules, such as ICAM-1 and VCAM-1 [157].

#### 4.3.2. Therapeutic Opportunities for MSCs Targeting the BBB in MS

The disruption of the BBB in MS allows immune cells to infiltrate into the CNS, contributing to the pathogenesis of MS, as previously mentioned. In vitro studies using a BBB model exposed to TNF-α have shown that treatment with embryonic MSCs modulates barrier permeability, increases the expression of TJ proteins, and decreases the expression of pro-inflammatory chemokines like CCL2 and CXCL12 [158] (Figure 4). In vivo studies have also demonstrated that co-administration of MSCs expressing IFN-β along with minocycline tames the disruption of the Blood-Spinal Cord Barrier (the functional equivalent of the BBB in the spinal cord) [159]. Recent studies in the MS preclinical model (experimental autoimmune encephalomyelitis) have further shown that MSC transplantation reduced BBB disruption, as evidenced by reduced IgG leakage. Additionally, MSC transplantation led to the adequate expression of TJ-proteins occluding and ZO-1 in BMVECs and the restoration of AQP4 levels in astrocytes [160].

### 4.4. Stroke

Stroke refers to an acute brain injury derived from no other than a vascular cause, leading to neuronal damage and functional disability. As a complex and heterogeneous condition, it is influenced by genetic predisposition, aging, life habits, and chronic diseases. It is the second leading cause of death and third leading cause of disability worldwide. Notably, 60% of surviving patients are affected by cognitive impairment, dementia, or depression. Stroke is classified into two types: hemorrhagic and ischemic, with the latter being the most prevalent (87% of the total). Ischemic stroke is characterized by a sudden cessation of oxygen and blood supply due to thrombus blocking blood flow in the brain vasculature [161].This initiates a rapid and complex cascade of pathophysiological events at the genomic, molecular, and cellular levels that may evolve over hours to days and weeks after the onset, including energy failure, acidosis, loss of cell homeostasis, excitotoxicity, oxidative stress, activation of glial cells, inflammation, and disruption of the BBB with infiltration of leukocytes [162,163].

#### 4.4.1. Dysfunctional BBB in Brain Ischemia

**Figure 5:**
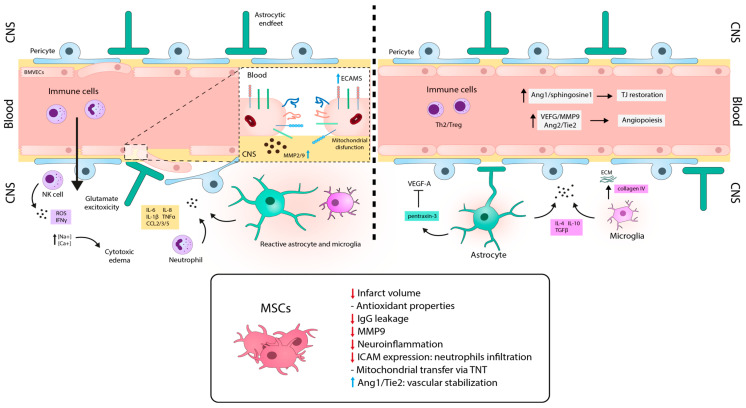
Scheme illustrating pathophysiology of blood-brain barrier permeability during stroke. Left panel, hyperacute and acute stages are characterized by endothelial cell destabilization and BBB disruption, edema, peripheral immune infiltration, and glial immune activation. Right panel, subacute and chronic stages are involved in the recovery phase. This is characterized by vascular organization, restoration of BBB integrity, and by anti-inflammatory and reparative immune activities. Treatment with MSCs during the hyperacute and acute phases reduces cytotoxic damage by the production of neurotrophic factors, by the decrease in the pro-inflammatory mediators and immune infiltration and by the recovery of BBB integrity. During the subacute and chronic stages, MSC treatment may collaborate with the endogenous mechanisms of recovery by keeping immunomodulatory properties and by favoring vascular stabilization. CNS: central nervous system; ECM: extracellular matrix; BMVECs: brain microvascular endothelial cells; ECAMs: endothelial cell adhesion molecules; NK: natural killer; MMP: metalloproteinases; VEGF: vascular endothelial growth factor; TJ: tight junctions; Ang: angiopoietin; Tie2: tyrosine kinase receptor. Blue and red arrows mean increase and reduction, respectively.

The BBB plays a significant role in the pathophysiology of ischemic stroke, and its dysfunction varies depending on the severity and duration of the ischemia. Predominantly, during human stroke, the BBB presents a continuous opening pattern with biphasic peaks distributed along four stages [164] (Figure 5). 

The hyperacute stage is the first phase that evolves within the first 6 h after the ischemic onset. During this phase, the first BBB opening is documented. Due to oxygen and glucose deprivation, Na^+^ and Ca^+^ accumulate inside the cells of the NVU, including astrocytes or BMVECs, leading to cytotoxic edema [165], glutamate excitotoxicity [166], oxidative damage associated with ROS generation, and mitochondrial dysfunction [164]. Moreover, MMPs (mainly MMP2) directly degrade TJ proteins and BM components, contributing to BBB leakage [167]. 

The next stage corresponds the acute phase, which occurs after the first 6 h of the onset for a period of 72–96 h. The second permeability peak is observed at this stage. From this point, immune components start to participate more significantly to stroke pathophysiology. Neutrophils are the predominant peripheral immune cells in the acute post-stroke period [168]. They contribute to BBB disruption by producing excessive ROS [169], proteases (MMP9, proteinase-3, elastase) [170], and neutrophil gelatinase-associated lipocalin [171], generating neutrophil extracellular traps [172], and secreting inflammatory cytokines (IL-1β, IL-6, IL-8, TNF-α) and chemokines (CCL2, CCL3, and CCL5) [173]. Moreover, P-selectin glycoprotein ligand-1 and macrophage-1 antigen in neutrophils, and their respective receptors, P-selectin and ICAM-1, in BMVECs, are upregulated shortly after an ischemic stroke induced by IL-1β. Hence, paracellular BBB permeability is increased [174,175]. Natural killer (NK) cells are also present during this stage, producing IFN-ϒ and ROS [176]. Monocytes and cerebral immune cells, microglia, and astrocytes display a pro-inflammatory phenotype which exacerbates BBB breakdown through the secretion of proinflammatory factors (IL-1α, IL-1β, IL-6, TNF-α, IFN-γ, CCL2), MMP9, VEGF, ROS, and activation of inducible nitric oxide synthase [162]. Finally, upregulation of MMP-9 is crucial in this phase (24–48 h after onset), and it plays a dual role in the proteolytic degradation of the BM components of the BBB. Its ability to digest TJ proteins enhances the disruption of the BBB [177]. Concomitantly, vascular remodeling is induced by VEGF, leading to the mobilization of progenitor BMVECs, which implies an immature and damaged BBB during this neovascularization process [178]. 

The subacute phase starts one week after the stoke onset, a timepoint from which the BBB begins its recovery process. Monocytes and glial cells shift to anti-inflammatory phenotypes expressing cytokines (IL-10, IL-4, TNF-β) and neurotrophic factors that help prevent inflammation [179]. Moreover, during this phase, angiogenesis plays a vital role in restoring the blood flow and oxygen supply in ischemic tissues. Angiogenesis promotes the proliferation and migration of BMVECs and pericytes, enhances tube formation, branching, and anastomosis, all of which are modulated by the inflammatory microenvironment. VEGF, MMP9, and angiopoietin 2 (Ang-2), as well as its receptor Tie-2, are deeply involved in this process [164]. While these agents may temporarily contribute to BBB leakage, a higher degree of angiogenesis has been linked to increased survival in patients [180] and greater stability of the BBB [181]. 

Finally, the chronic post stroke phase starts approximately six weeks after the ischemic event, where the BBB is still disrupted but to a lesser extent compared to the previous phases. During this stage, NVU components are restored in order to seal the BBB, with an overexpression and new distribution of TJ proteins. Some of the factors that stabilize the BBB include Ang-1, which maintains BMVECs in a quiescent state and contributes to junction generation, and sphingosine-1 phosphate and activated protein C, which help balance junction proteins and cytoskeleton [182]. Immune cells also shift to anti-inflammatory phenotypes and develop mechanisms to diminish BBB breakdown. For example, anti-inflammatory astrocytes release pentraxin-3, which inhibits VEGF in the ischemic cerebral tissue and specifically supports BBB integrity [183]. Finally, neuronal progenitor cells migrate to the ischemic tissue to favor neurogenesis and neuroplasticity and restore BBB components [184,185].

#### 4.4.2. Therapeutic Opportunities for MSCs Targeting the BBB in Brain Ischemia

Several studies have demonstrated the therapeutic effect of MSCs in preclinical models of stroke (Figure 5). Thus, understanding their effect on the BBB has become a topic of great interest in research [186]. 

For example, MSCs have shown the ability to protect neurons against oxidative damage. A study conducted by Huang et al. [187] showed that MSCs express high levels of antioxidant enzymes from the peroxiredoxin (PRDX) family. In this report, MSCs were able to rescue BBB integrity in an in vitro model of oxidative damage with the bEnd.3 cell line, by reducing TJ degradation and excessive ROS generation. This effect was found to be partially mediated by the secretion of PRDX4. Interestingly, silencing PRDX4 in MSCs attenuated their protective effect on BBB integrity, both in vitro and in the in vivo MCAO ischemic model. Conversely, Cheng et al. [188] investigated the effect of the treatment of MSCs administered 15 min after MCAO and found a reduction in IgG leakage in the brain parenchyma, along with the reversal of the TJ protein gap formation (ZO-1, occludin, and claudin-5). This treatment also suppressed MMP-9 upregulation, reduced neuroinflammation, and decreased neutrophil infiltration with downregulation of ICAM-1 expression. In a related study, Liu et al. [189] demonstrated that co-culture of MSCs with HUVECs in an in vitro ischemic-reperfusion model rescued injured endothelial cells via the generation of TNT-like structures, which was dependent on F-actin polymerization. They further investigated this effect in vivo in a model of MCAO in rats [190], in which the injection of MSCs 24 h after the ischemic induction led to a significant reduction in the infarct volume and higher microvessel densities in the peri-infarct areas. This was attributed to rescuing brain microvasculature through mitochondrial transfer with TNT formation in vivo. Moreover, Zacharek et al. [191] demonstrated that the administration of MSCs 24 h after MCAO in rats reduced BBB leakage and promoted angiogenesis and vascular stabilization in vivo and in vitro. This effect was achieved by increasing endogenous Ang-1/Tie2 and TJ proteins, and promoting the crosstalk between BMVECs and astrocytes. 

Finally, different studies have demonstrated that the administration MSC-EVs can lead to a reduction in the infarct volume, improve neurological recovery, and enhanced angiogenesis. These effects are particularly significant when MSCs are exposed to hypoxic conditions prior to EV isolation [192]. These results suggest that it is not necessary to use the whole MSC for the treatment of ischemic stroke [193,194], evidencing MSC-EVs as a promising alternative for stroke therapy. Overall, these studies suggest that MSC treatment has a significant impact on BBB integrity and a great potential to modulate microvasculature and reduce the pathological processes associated with stroke. 

## 5. Challenges and Future Directions

While preclinical studies have yielded promising results regarding the therapeutic potential of MSCs in neurodegenerative and neuroinflammatory disorders, the clinical efficacy of using MSCs remains uncertain and has produced mixed results (Table 2). A recent meta-analysis conducted by Kvistad et al. [195] aimed to assess the safety and efficacy of MSC therapy in various neuroinflammatory conditions, encompassing MS, ischemic stroke, and traumatic spinal cord injury. The findings indicated that the treatment was generally safe and well-tolerated by patients. However, the efficacy results were inconclusive, with no significant improvements. Irrespective of the neurodegenerative conditions discussed in this review, the limited efficacy observed in MSC treatment and the disparities between outcomes in mouse models and human clinical trials may be attributed to several factors. These include the specific source, culture, and isolation methods employed for MSCs, the dosage of transplanted cells, and the timing and route of delivery [195,196,197,198,199,200]. Furthermore, the accurate selection of patients and the identification of outcome measurements for assessing the success of MSC treatment are critical considerations that can significantly contribute to differences observed in clinical trials.

- Source and culture of MSCs: MSCs from different sources such as BM-MSCs, ASCs, neural stem cells, and umbilical cells have been used in clinical trials (from both autologous and allogeneic origins), generating different results. These variations can be partially attributed to their distinct paracrine functions, leading to the secretion of different angiogenic, growth, and cytokine factors, which in turn influence their neuroprotective and immunological capabilities [201,202]. Furthermore, the methods used to harvest and isolate different MSCs can impact their yield, viability, and differentiation potential [203,204]. Depending on the source and intended use of the (allogeneic versus autologous) MSCs, the need of an extensive culturing process can also increase the senescence of MSCs, ultimately affecting the proliferative rates and their therapeutic efficacy [205,206]. Thus, significant differences exist in the methodological approaches used in the culture of MSCs, including culture reagents, cell expansion, cryopreservation, thawing procedures, fitness assessments, and functionality evaluations. As a result, there is a need for standardized protocols in the laboratory management of MSCs to mitigate inconsistencies across studies. In fact, the majority of human clinical trials employ allogeneic cryobanked MSCs, which are thawed immediately prior to transfusion. Cryopreservation methods and thawing protocols can also contribute to variations among clinical studies [207]. It is important to note that MSCs display molecular signs of cell injury in the first 24 h following retrieval from cryostorage. These molecular changes correlate with defects in suppression function in vitro, increased susceptibility to immune cell lysis, as well as reduced persistence in vivo following intravenous transfusion [208]. Allogeneic human MSCs typically transfused into patients within a few hours post thaw, directly retrieved from cryostorage, probably exhibit reduced viability, functionality, and in vivo persistence, compared to the cells routinely used in analogous murine systems. Additionally, patient-specific factors such as age, gender, genetic traits, existing co-morbidities, and the systemic effects of medications must be considered in autologous MSC treatments [197,209]. Interestingly, while autologous MSCs have been extensively used in most of the trials for AD, PD, and MS, their prolonged culture times needed for cell expansion make them less suitable for acute tissue injury conditions like stroke. In such conditions, allogeneic MSCs become the more feasible strategy [210]. Specifically, the clinical trials using allogeneic ASCs against neurodegenerative disorders have significantly increased in the last years (Table 2). These cells are abundant in adults and easy to isolate and expand. Furthermore, ASCs offer advantages for allogenic treatments, as they can be used to create a standardized and cost-effective donor bank, avoiding the issues associated with autologous treatments such as donor-recipient compatibility. However, it is important to note that during in vitro expansion major HLA class II molecules may increase in expression [211]. Allogeneic MSCs, when exposed to serum, can be vulnerable to complement-mediated injury, leading to reduced viability after infusion compared to autologous MSCs [212]. Therefore, in scenarios where long-term repeated administrations are necessary to impact outcomes for chronic disorders, the examination of immunological compatibility between donor MSCs and recipients may extend the survival and effectiveness of the MSCs [213].
- MSC dose, timing and delivery: The challenges and disparities observed in clinical trials are also closely related to significant differences in cell doses and transplantation timing between laboratory settings and clinical practice. For instance, in preclinical studies involving stroke, an effective intravenous dose typically amounts to around four million cells for a rat weighing 250 g. This would translate to approximately 840 million cells in a stroke patient weighing 75 kg [214]. However, most clinical trials use doses considerably below this efficacious threshold, which may explain the observed lack of efficacy [215,216,217]. It is worth noting that stroke patients who received doses aligned with the findings of these preclinical studies displayed clinical improvements [218]. In addition, considerations regarding the administration of single or multiple doses, as well as the timing of these injections, can also result in significant differences in outcomes. Standardization of dosage is crucial to reduce variability between trials and gain insights into areas that require improvement. Additionally, the optimal route for stem cell injection can vary depending on the nature of the disease. For instance, in the case of MS, which is a multifocal and systemic disease, intravenous administration offers a straightforward and safe way to modulate aggressive immune responses in peripheral lymphatic tissues. A more localized distribution is desirable in conditions like PD, AD, and stroke after MSC delivery. However, in all of these disorders, despite the benefits observed when MSCs are intravenously injected in animal models, less successful therapeutic outcomes are often observed in clinical settings. This discrepancy is likely due to the entrapment of MSCs in systemic organs and the challenges associated with transmigration across the blood-brain barrier [219,220]. While intra-arterial administration of MSCs could serve as an alternative to intravenous administration, this approach increases the risk of embolic events [221,222]. The procoagulation status associated with MSCs and their size, which can lead to their arrest in small-diameter vessels, potentially causing vascular occlusion and reduced cerebral blood flow, may explain the risk of lethal pulmonary thromboembolism [223]. On the other hand, direct injection via intrathecal or intraventricular routes has shown promise [224,225]. While no significant differences were detected between intravenous and intrathecal routes in terms of MSC efficacy in patients with conditions like MS [226], some studies suggest that intrathecal administration of MSCs may be more efficacious than the intravenous route [227,228]. An alternative non-invasive and rapid delivery route for treating CNS disorders is intranasal administration of MSCs. Finally, preclinical models have demonstrated successful delivery of MSCs to the brain through this route, with therapeutic efficacy observed in conditions such as PD, MS, AD, and stroke [229,230,231,232].
- Selection of patients: A significant source of variability in the outcomes of clinical trials stems from the heterogeneity of patient populations. This heterogeneity encompasses factors such as disease onset, development, stages (in AD, PD, and MS), and pathological phases of the disease (in stroke), disease severity, and the presence of co-morbidities. Therefore, careful consideration must be given to the rational selection of optimal candidate patients. For instance, in conditions like stroke, different phases of the pathological ischemic process offer distinct targets for MSC therapy, with varying beneficial effects. Early-phase trials have shown that MSC administration in stroke patients is safe and can reduce the inflammatory response, regulate the dynamic environment against toxicity, and decrease injury in the peri-infarct area [233,234]. However, the effectiveness of MSC therapy in clinically subacute and chronic ischemic stroke cases is yet to be fully validated. In MS, stem cell transplantation is more efficient when performed in the early stages of the disease because, in the later phases, after the emergence of chronic lesions, endogenous repair processes become compromised [235]. While endogenous repair mechanisms and injected MSCs can work in synergy during the repair of active plaques in relapsing-remitting MS, progressive forms of the disease witness a reduction in the function of reparative cells due to gliosis and the formation of fibrotic scars [236]. Similarly, in AD and PD, despite promising preclinical data, most clinical trials involving MSCs have yielded disappointing results due to the pathological variability corresponding to different stages of the diseases [196,198]. This heterogeneity in disease stages and phases introduces considerable variability across trials, both in terms of the therapeutic windows (spanning from acute to chronic stroke stages) and the routes of administration [215,216,217,237,238,239]. To address these challenges, the development of specific animal models with interventions tailored to particular disease stages will be essential for the effective clinical translation of novel therapeutics.
- Clinical outcomes: Developing uniform and standardized measurements for assessing the efficacy of stem cell therapy is indeed crucial for reducing clinical failure. In diseases like PD and AD, where early-stage diagnostic tests are lacking, disease identification is often based on symptoms that manifest in the later stages, when a substantial portion of neurons has already been damaged, or through post-mortem pathological examinations. Consequently, the classification and staging of these diseases can vary significantly, leading to a high degree of heterogeneity in defined clinical groups. Indeed, achieving a more objective and standardized approach to patient selection and treatment evaluation is essential. This can be facilitated by the incorporation of various objective measures, including functional magnetic resonance imaging (fMRI), magnetic resonance tractography, and the use of blood and cerebral fluid biomarkers [240,241]. These measures not only may aid in identifying clinical improvements but also contribute to a more accurate assessment of disease progression and response to treatment. Furthermore, the timing of follow-up assessments is another critical consideration. Using more uniform clinical grouping criteria and outcome measures is pivotal for effectively evaluating the outcomes of cell therapy treatments. By adopting standardized assessment tools and criteria, researchers and clinicians can better compare results across different trials and refine treatment strategies for improved patient outcomes.

In summary, the valuable insights gained from clinical trials should guide future directions in stem cell therapy and underscore the critical need for a systematic approach to identify optimal conditions that can yield reliable and effective treatments. Several key strategies can contribute to achieving this goal, such as: (i) developing live imaging techniques to track and assess the bioavailability and biodistribution of MSCs in the body can provide valuable real-time information about their behavior and interactions; (ii) standardizing isolation and expansion protocols for MSCs that will help to reduce variability and ensure consistent quality in cell manufacturing; (iii) the incorporation of clear and reliable biomarkers that accurately reflect the therapeutic effects of stem cells in patients and thus can provide objective measures of treatment efficacy; and (iv) conducting well-designed randomized controlled studies that will help to establish the safety and efficacy of stem cell-based therapies while minimizing bias and confounding factors. To address the challenges and improve the clinical outcomes of MSC therapy in neuroinflammatory pathologies, it is imperative to gain a clearer understanding of the underlying biology and mechanisms of action of MSCs as well as to identify new cellular and/or molecular targets involved in these diseases. This knowledge will serve as a foundation for optimizing the treatment approach and developing targeted strategies that can enhance therapeutic efficacy. Regarding this, MSC priming or preconditioning approaches that expose cells to growth conditions mimicking the in vivo microenvironment of damaged tissue can improve the efficiency of MSC-mediated therapy. Various priming methods, such as using inflammatory cytokines, hypoxia, pharmacological drugs, chemical agents, biomaterials, and specific culture conditions, have shown promise [242]. However, it is important to note that the challenge lies in achieving consensus on cell manufacturing protocols for priming, which can ensure quality assurance for clinical-grade MSCs [243]. Additionally, as discussed in this review, the therapeutic potential of MSCs extends beyond their homing and differentiation capabilities. Their paracrine secretion of various factors, including trophic factors, immunomodulatory molecules, microvesicles, microRNAs, and mitochondrial transfer, plays a central role in their therapeutic effects. Recent investigations have explored cell-free therapies as promising approaches in neurodegeneration. Studies have shown that systemic administration of MSC-EVs can lead to functional improvement, offering a similar functional outcome with an improved safety profile compared to MSC administration, particularly in preclinical stroke models [244]. The use of allogeneic MSC-EVs confers several advantages, including low toxicity, low immunogenicity, enhanced safety, ease of injection, and improved ability to trespass biological barriers avoiding undesired entrapment in the lung microvasculature, and advantages in scalable production and storage [245]. However, despite promising preclinical results, only a few clinical studies on the effects of EV therapy in humans have been reported. These studies, while limited in scale, suggest that MSC-EVs are safe and may improve patient outcomes. Nevertheless, larger randomized trials are needed to thoroughly investigate the efficacy and safety of MSC-EVs therapy [246,247,248]. It is important to note that the cargo contents of MSC-EVs may exhibit age-dependent differences [249]. Several critical considerations must be addressed before the widespread clinical application of EVs. These include the establishment of specific guidelines for EV-based therapeutics, standardization of EV characterization, isolation, and storage methods, quality control requirements, and in-depth in vivo analyses of EV functionality. Variability in EV functional properties arising from different culture conditions is a crucial factor to consider for successful clinical translation.

Finally, recognizing that MSCs not only impact immune activation and neurodegeneration but also address disrupted vascular components has prompted the exploration of novel therapeutic targets and combination strategies. In addition to their impact on the BBB in the context of the CNS disorders covered in this review (PD, AD, MS, and stroke), the relevance of the BBB extends to other neurodegenerative conditions, including traumatic brain and spinal cord injury [250,251,252], amyotrophic lateral sclerosis [253,254], Huntington’s disease [255], and epilepsy [256]. However, the significance of the BBB as a target for MSC therapy in those disorders is still emerging. The understanding of the role of the BBB in the pathophysiology of neurodegenerative and neuroinflammatory diseases is pivotal for developing multi-target-based approaches. As discussed, the ability of MSCs to restore BBB integrity and functionality in aging-associated CNS pathologies such as AD, PD, MS, and stroke combined with their capacity to modulate the immune response and neurodegeneration, positions them as an attractive therapeutic option. This opens up the possibility of extending BBB targeting by MSC therapy to include other CNS-injured conditions. Prioritizing the BBB in the development of MSC-based therapies holds promise for enhancing clinical outcomes for patients suffering from these and other debilitating conditions.

## Figures and Tables

**Table 2:** Mesenchymal Stromal Cell-Studies selected from ClinicalTrials.gov for “Multiple Sclerosis”, “Ischemic Stroke”, “Alzheimer disease”, and “Parkinson disease”.

Components in the Clinical Trials	Categories	Studies (%)
MSC type	Bone Marrow	27 (30)
	Umbilical Cord	24 (26.67)
	Adipose	14 (15.56)
	Neural Progenitor-derived	4 (4.44)
	Embryonic	1 (1.11)
	Exosomes	1 (1.11)
	Not indicated	20 (20.22)
Disorders/Conditions	Multiple Sclerosis	35 (38.89)
	Ischemic Stroke	25 (27.78)
	Alzheimer	17 (18.89)
	Parkinson	13 (14.44)
Modality	Autologous	41 (45.56)
	Allogenic	21 (23.33)
	Not indicated	28 (31.11)
Route	Intravenous	48 (53.33)
	Intrathecal	8 (8.89)
	Intravenous/Intrathecal	3 (3.33)
	Intraventricular	1 (1.11)
	Intra-striatal	1 (1.11)
	Intracerebral	1 (1.11)
	Nasal	1 (1.11)
	Not indicated	27 (30)
Target	Score	76 (76.77)
	Immune	13 (13.13)
	Neurological	10 (10.10)

## Data Availability

Not applicable.

## Abbreviations

The following abbreviations are used in this manuscript:

AJs	Adherens junctions
ASCs	Adipose tissue-derived MSCs
AD	Alzheimer’s disease
BM	Basement membrane
BBB	Blood-brain barrier
BM-MSCs	Bone marrow-derived mesenchymal stem cells
BMVECs	Brain microvascular endothelial cells
CNS	Central nervous system
CCL2	Chemokine (C-C motif) ligand 2
Aβ	Extracellular amyloid-β
ICAM1	Intercellular adhesion molecule 1
IL	Interleukin
MSC	Mesenchymal Stem Cells
MSC-EVs	MSC-derived extracellular vesicles
MCAO	middle cerebral artery occlusion
MS	Multiple sclerosis
NGF	Nerve growth factor
NVU	Neurovascular unit
PD	Parkinson’s disease
PRDX	Peroxiredoxin
PPMS/SPMS	Primary/secondary progressive MS
RRMS	Relapsing-remitting MS
sRAGE	Secreted isoform of RAGE
SNpc	Substantia nigra pars compacta
TJs	Tight junctions
TIMPs	Tissue inhibitor metalloproteinases
VEGF	Vascular endothelial growth factor
WJ	Wharton’s jelly
